# The Attribution of Mental Health Problems to Jinn: An Explorative Study in a Transcultural Psychiatric Outpatient Clinic

**DOI:** 10.3389/fpsyt.2018.00089

**Published:** 2018-03-28

**Authors:** Anastasia Lim, Hans W. Hoek, Samrad Ghane, Mathijs Deen, Jan Dirk Blom

**Affiliations:** ^1^i-psy Intercultural Psychiatry, Parnassia Psychiatric Institute, Utrecht, Netherlands; ^2^Parnassia Academy, Parnassia Psychiatric Institute, The Hague, Netherlands; ^3^Faculty of Social and Behavioural Sciences, Leiden University, Leiden, Netherlands; ^4^Dutch Institute of Forensic Psychiatry and Psychology, Utrecht, Netherlands; ^5^Department of Psychiatry, University of Groningen, Groningen, Netherlands; ^6^Department of Psychiatric Epidemiology, Columbia University, New York, NY, United States; ^7^Arq Psychotrauma Expert Group, Equator Foundation, Diemen, Netherlands

**Keywords:** cultural concept of distress, hallucination, idiom of distress, Islam, Muslim

## Abstract

**Background:**

Among Muslim patients, a common cultural concept of distress is the notion that jinn may be the cause of mental health problems, especially in the presence of hallucinations.

**Objective:**

This study examines the frequency with which this attribution style is manifest in a specific psychiatric outpatient population with a Muslim background.

**Methods:**

Of all patients registered at an outpatient clinic specialized in transcultural psychiatry, data were collected on folk belief, religion, hallucinations (if present), and medical diagnosis. Through a search in the electronic medical files, the notes made during the first contact and first psychiatric examination were screened for the keywords “evil eye,” “magic,” “voodoo,” and “jinn.” In addition, new eligible cases were accepted.

**Results:**

From all 551 patients thus screened, 118 were eligible for participation. Of these, 49 (41.5%) were interviewed using a semi-structured questionnaire. Among them, 21 (43%) were positive that their psychiatric symptoms were caused by jinn, whereas 13 (27%) thought not, and 15 (31%) were in doubt. No less than 87.2% had experienced hallucinations during their lives. Among the relatively large proportion of eligible patients who did not participate (58.5%), many expressed a fear for stigmatization or metaphysical repercussions if they spoke about jinn.

**Conclusion:**

The phenomenon of attributing mental health symptoms to jinn was much more common in this population of Muslim patients than previously assumed. This underscores the need for proper knowledge of Muslim explanatory models of disease and for the use of culturally sensitive interviewing techniques in this population.

## Introduction

The quality of mental health care for patients with a Muslim background is a timely issue for Western practitioners, researchers, and policymakers (1–4). Faced with an explanatory model that often differs markedly from that of other patients, biomedically trained health professionals may encounter difficulties with this group when trying to establish a diagnosis and making an appropriate treatment proposal. Therefore, it is essential to have adequate background knowledge of the attribution styles frequently used by Muslim patients (5–7). Although many of these patients may strongly endorse Western biomedical thinking, others may more readily attribute their symptoms to the evil eye, magic (voodoo), or jinn (1, 8, 9). Many of these beliefs are rooted in pre-Islamic traditions and folk belief (10), and for jinn, it is partly rooted in the official Islam. In daily life, the majority of non-orthodox Muslims can adhere to either of these discourses or to an idiosyncratic mixture of beliefs, whether or not in combination with a belief in spirit possession, yet another important (and partially overlapping) discourse (11).

As biomedically trained health professionals tend to use a conceptual framework that precludes explanations in culturo-religious terms, patients may feel misunderstood or misrepresented (12). Conversely, health professionals may either be ignorant of the heuristic gap they are dealing with or, being aware of it, feel powerless to overcome it (13, 14). In either case, patients may fail to benefit from the treatment programs offered, or may face unnecessary delays while trying to gain access to them (15).

Another important factor in the encounter with Muslim patients in Western consulting rooms is that many of them have a personal migration background and, on top of that, some have had first-hand experience with traumatizing events in war zones (2). However, even in the absence of war-related trauma, studies indicate that migration itself is an important risk factor for psychiatric disorders, including posttraumatic stress disorder (PTSD), and dissociation (16). This is especially the case when migrants face a substantial cultural gap between their country of origin and that of their destination. For example, Moroccans in the Netherlands, who usually do not originate from conflict areas, but need to bridge a wide cultural gap, have been consistently found to have an elevated risk for developing mental disorders, notably schizophrenia spectrum disorders (17–19). In addition, low socioeconomic status, language barriers, feelings of shame, and (self-)stigmatization, as well as fear for supernatural repercussions, may contribute to a delayed trajectory toward adequate care (20, 21).

In the Netherlands (with a population of 17 million), approximately 5% of the inhabitants are Muslims (22, 23). Of these, the majority are Turkish (*n* = 397,471) or Moroccan (*n* = 385,761) (23). From our clinical practice and the related literature, it is known that patients from these and other Muslim groups are inclined to attribute problems in life, including mental health problems, to jinn (1, 24).

In the Qur’an, jinn are described as beings created by Allah out of smokeless fire (Qur’an; 15: 26-27). They are mentioned in 29 different places, including the chapter entitled *Al-Jinn* and one about King Solomon. In the Hadith (sayings of the Prophet), jinn play an even more prominent role, although they are described most evocatively in Islamic folk belief. On the basis of these sources, jinn are conceptualized as beings normally invisible to the human eye, but capable of making themselves visible if they so wish and also capable of interfering with humans in powerful ways. Thus, they are endowed with the ability to travel great distances with incredible speed, to eavesdrop on humans and supernatural beings, to move through concrete walls, to appear in the shape of various animals, to take possession of humans, and to marry humans in secret to thus prevent them from entering a human relationship. As they are also said to be easily offended and to live much longer than humans, they are considered capable of settling old scores with those who offended them, bridging successive generations. Because of this unnervingly unequal power balance and the fact that the existence of jinn is part and parcel of the Islamic world view, in many Muslims, these beings spring to mind whenever something goes wrong (25) even though jinn are also considered capable of aiding humans to attain positive goals in life (4, 26, 27).

Although insight into the belief in jinn as part of Muslim explanatory models of disease has grown over the years (1), it is still in its infancy. Much of the existing literature consists of case reports written from the vantage point of Western physicians and accounts of a more expository nature published by Western medical anthropologists. Based on studies such as these, it is impossible to establish whether we are dealing with a remarkable, yet relatively rare attribution style, or whether the reported cases of patients attributing their mental health problems to jinn are the tip of the iceberg.

Therefore, the present study investigates the frequency of this occurrence by (i) interviewing Muslim patients attending an urban outpatient clinic specialized in transcultural psychiatry and (ii) collecting data on their religious background and their experiences with hallucinations, including (if applicable) the phenomenological characteristics of those hallucinations.

## Materials and Methods

### Participants

At the transcultural outpatient clinic i-psy, Parnassia Psychiatric Institute (Utrecht, the Netherlands), Muslim patients were recruited who were thought to attribute their mental health problems to a supernatural cause. Inclusion criteria were age ≥18 years, Muslim background, and willingness to give informed consent. After oral and written explanation of the study, all the participating patients gave informed consent.

### Instruments

A search was made in patients’ electronic medical files (covering the period April 2013 through January 2015). To identify patients familiar with Muslim concepts of distress, we screened the notes made at first contact and those made during the first psychiatric examination for the keywords “jinn,” “evil eye,” “magic,” and “voodoo.” Whenever such terms were found, the relevant patients were invited to participate in the present study. In addition, we requested all health professionals at the outpatient clinic to screen their caseloads for eligible patients. During the course of the study, new eligible cases were also allowed to enroll. Demographic data, comprising gender, date of birth, country of birth, ethnicity, migration history, socioeconomic status, and level of education, were derived from the patient files and interviews. The interviews were all conducted by psychiatrists, psychiatric residents, psychologists, and psychologists in training, who had been trained to use the Hallucination Attribution List, a tailor-made semi-structured interview for Muslim patients that focuses on religion, attribution style, and (if applicable) the presence and phenomenological characteristics of hallucinations. The presence and (if applicable) phenomenological characteristics of hallucinations were explored in depth to chart the sensory modalities in which they were experienced, their frequency, and their duration. Certain items were rated “yes” or “no,” others were measured on a scale from 0 to 100. All psychiatric diagnoses at the start of treatment were made or confirmed by the patients’ treating psychiatrists, in conformity with the fourth edition of the Diagnostic and Statistical Manual of Mental Disorders [DSM-IV-TR (28)]. Whenever patients were insufficiently fluent in Dutch, an independent interpreter was hired at the Center for Interpreters to conduct the interview, in collaboration with one of the researchers.

### Statistical Analyses

Data were analyzed with Fisher’s exact test and Kruskal–Wallis test, using SPSS 23.0 and Microsoft Excel 2010.

## Results

**Figure 1:**
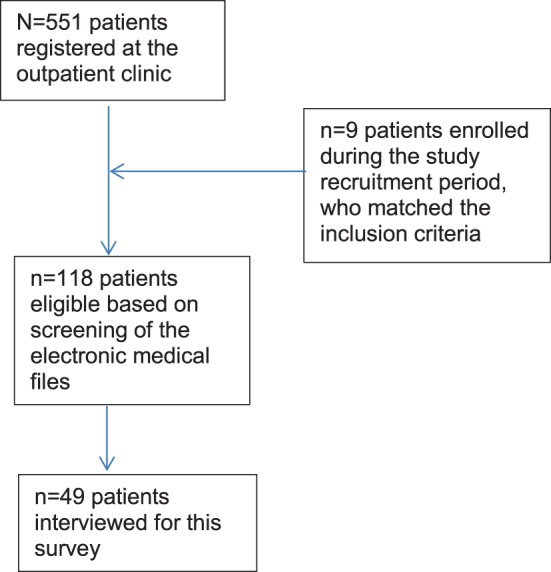
Flowchart of patient participation.

At the start of this study, 551 patients aged ≥18 years were registered at the outpatient clinic. Of these, 109 (19.8%) were considered eligible for participation (Figure 1). During the course of the inclusion phase, nine additional participants were enrolled. Of the resulting 118 eligible patients, 49 (41.5%) gave informed consent and were subsequently interviewed. However, three of these participants were unable to complete the interviews because they felt unwell and wished to terminate the session due to fear for aggravation of their symptoms; nevertheless, these cases were included in the analysis because (even though incomplete) they were considered to be informative and valuable. The 69 patients who did not wish to participate either (i) had finished their treatment by the time they were invited, (ii) were afraid of the possible consequences they might suffer when talking about jinn and related subjects, (iii) had dropped out of the treatment program, (iv) were psychiatrically too unstable to participate, or (v) were not available due to holidays or the Ramadan.

### Demographics

Of the 49 patients who participated in the interviews, 27 were men (55.1%). Age ranged from 24 to 59 (mean 43.9, SD 9.42) years. Most patients were born outside the Netherlands (*n* = 43; 87.8%) and were therefore considered first-generation immigrants. The majority were originally from Turkey (40.8%) or Morocco (40.8%) and the remainder were from Afghanistan (4.1%), Iran, Iraq, Syria, Lebanon, Palestine, and Guinea (each 2%). More than half of the patients were sufficiently fluent in Dutch to be interviewed without an interpreter (*n* = 28; 57.1%), whereas 16 were not sufficiently fluent (37.7%) and five spoke no Dutch at all (10.2%). These latter 21 patients were interviewed with the aid of an interpreter, either face to face or by telephone. Almost one-third of the group (32.7%) had been educated at an elementary school, while a similar proportion had also finished high school (32.7%). A minority (8.2%) had finished community college, and a slightly larger group (12.3%) college or university. Of all interviewees, 14.3% had received no formal education at all. No association was found between the attribution of symptoms to jinn and (i) the level of education (Kruskal–Wallis test: *p* = 0.21), (ii) ethnicity (Fisher’s exact test: *p* = 0.053), or (iii) the presence of a language barrier (Kruskal–Wallis test: *p* = 0.30).

### Diagnosis

In this study population, the vast majority of patients had been diagnosed with a mood disorder (55.1%), PTSD (12.2%), or other anxiety disorder (12.1%). Only 8% of the patients met the diagnostic criteria for schizophrenia or a related disorder. The remainder of the group (10.2%) had diverse diagnoses, i.e., body dysmorphic disorder (*n* = 1), schizotypal personality disorder (*n* = 1), acute stress disorder (*n* = 1), adjustment disorder (*n* = 1), and post-concussion syndrome (*n* = 1). In one medical file, the initial diagnosis was missing (2%). Because of the relatively small numbers, we were unable to establish any correlations between diagnosis and attribution style.

### Religion and Attribution Style

Although all participants had a Muslim background, of the 49 interviewees, 2 had converted to Christianity. In conformity with their new-found faith, both these participants stated that they did *not* attribute any of their symptoms to jinn, black magic, or the evil eye. Of the remaining group of 47 Muslim interviewees, 80.9% (*n* = 38) believed in the evil eye, 63.8% (*n* = 30) in magic, and 78.7% (*n* = 37) in jinn (Table 1). Moreover, 57% (*n* = 27) claimed to have had first-hand experiences with one or more jinn in the past, versus 23.4% (*n* = 11) who denied any personal encounters and nine participants (19.1%) who expressed their doubts about this. When asked whether it is possible that their psychiatric symptoms were caused by these metaphysical beings, in the group of interviewees, 44.7% (*n* = 21) confirmed this, 23% (*n* = 11) were convinced that this was not the case, and the remaining 32% (*n* = 15) expressed doubts as to whether jinn had anything to do with their mental health problems, although they admitted that they had considered the possibility (Table 2). One participant was so overwhelmed that it was not possible to explore the theme in any more depth.

**Table 1:** Attribution styles (*n* = 47).

	Belief in Jinn (%)	Magic/voodoo (%)	Evil eye (%)
Yes	37 (78.7)	30 (63.8)	38 (80.9)
No	6 (12.8)	6 (12.8)	5 (10.6)
In doubt	4 (8.5)	9 (19.1)	3 (6.4)
Missing	–	2 (4.3)	1 (2.1)

**Table 2:** Attribution of mental health symptoms to jinn (*n* = 47).

	Attribution to Jinn (%)	Alternative, if not jinn	Alternative, if not jinn
		
		Magic/voodoo (%)	Evil eye (%)
Yes/possible	21 (44.7)	10 (21.3)	6 (12.8)
No/improbable	11 (23.4)	22 (46.8)	25 (53.2)
In doubt	15 (31.9)	8 (17.0)	10 (21.3)
Missing	–	7 (14.9)	6 (12.8)

### Symptoms

When exploring the symptoms further, the participants mentioned anxiety, obsessive-compulsive symptoms, aggression attacks, nightmares, physical pain, sadness, suicidal thoughts, weakening of the legs, headaches, insomnia, brooding, flashbacks of traumatic events, incubus phenomena, sensed presence, and hallucinations. The two patients who had been converted to Christianity reported hallucinations (one auditory, and one auditory and visual), insomnia, anxiety, and posttraumatic stress. In the group of 47 Muslim patients, these widely varying symptoms were attributed with great certainty to jinn by 14.8% of the participants (*n* = 7; i.e., *n* = 1 aggression attacks; *n* = 3 verbal auditory hallucinations; *n* = 1 depression; *n* = 1 dissociation and depression; *n* = 1 insomnia). Although 19 patients (38.7%) were doubtful about this, they did not dare to dismiss the possibility. The incubus phenomenon (i.e., sleep paralysis combined with the sensation of a metaphysical being sitting on the thorax and producing feelings of suffocation and panic) was described by 23.4% (*n* = 11), while sensed presence (i.e., the experience of some person or entity being present while there is no one around) was described by 21.2% (*n* = 10). Of the latter patients, three were convinced that jinn were to blame, versus two from the incubus group. In both groups, however, 11% said that jinn *might* be the cause.

Of the 47 participants, 41 (87.2%) had experienced hallucinations at least once during their lives: of these, 34% (*n* = 16) had experienced unimodal hallucinations, including one person who described a typical hypnagogic hallucination and one who reported visual hallucinations under the influence of morphine during postoperative recovery. In all other cases, participants experienced hallucinations in the auditory (19.1%, *n* = 9), visual (8.5%, *n* = 4), or tactile modalities (2%, *n* = 1). No participants had had any unimodal olfactory or gustatory hallucinations, whereas 25 (53.2%) had experienced hallucinations in multiple sensory modalities (two modalities *n* = 17; three modalities *n* = 6; four modalities *n* = 2). In the case of two modalities, most had experienced auditory and visual hallucinations (29.8%, *n* = 14), whereas the others had experienced hallucinations in the auditory and tactile (*n* = 1), visual and tactile (*n* = 1), or auditory and gustatory (*n* = 1) modalities. In this latter group, none of the participants had experienced hallucinations in these sensory modalities simultaneously, meaning that they had experienced multimodal hallucinations, not compound hallucinations. In the group of three sensory modalities, five participants had experienced hallucinations in the auditory, visual, and tactile modalities; one of them had eventually been diagnosed with multiple sclerosis. One of the other hallucinating patients had also experienced derealization and micropsia (e.g., seeing her husband’s body shrink, seeing a dwarf walking through the room), i.e., symptoms characteristic of an Alice in Wonderland syndrome (29). One participant had experienced hallucinations in the auditory, visual, and olfactory modalities. The two participants who had reported hallucinations in four sensory modalities had experienced them in the auditory, visual, gustatory, and olfactory modalities and in the auditory, visual, tactile, and olfactory modalities, respectively. Of the participants who had experienced only unimodal hallucinations, the majority doubted whether these were caused by jinn, whereas all participants with hallucinations in three or four sensory modalities were (almost) certain about this. The two participants with hallucinations in four sensory modalities were entirely certain that jinn were the cause of their symptoms. Statistically, however, we found no significant associations between the presence of hallucinations in various sensory modalities and the attribution of these phenomena to jinn. The same held true for the certainty with which participants attributed their symptoms to jinn.

## Discussion

In this transcultural psychiatric outpatient population, we investigated how often Muslim patients attributed their mental health problems to jinn. In the group of interviewed patients, 44.7% stated that their psychiatric symptoms could be caused by jinn. Looking at the total eligible group of 118 patients, the percentage of attribution was only 17.7% (*n* = 21 of 118 patients). We estimate that this number might have been higher had all eligible patients agreed to participate. An earlier investigation of this attribution style in a clinical population of psychotic patients was estimated at 80% (24). However, it should be noted that the latter patient group differs substantially from the present group, in that all the latter patients were diagnosed with a schizophrenia spectrum disorder and were so severely ill that admission had been necessary (in some cases under the Dutch equivalent of the Mental Health Act). Nevertheless, the findings in the present outpatient group are expected to be an underestimation of the actual situation, since 58.5% of the patients eligible for participation (69 of 118) decided to back out. Some of these patients openly admitted that they were afraid of repercussions from jinn if they would speak about them, whereas others considered it inappropriate to discuss issues such as these with a non-Muslim, or denied (contrary to written evidence in their records) that they had ever thought about the subject or even knew what jinn might be. In the light of these reactions, it is also conceivable that, *vice versa*, we may have missed eligible patients during the search of the electronic medical files because they had never discussed their suspicions regarding the role of jinn with their treating mental health professionals. In general, minority groups are hard to recruit for scientific research (30). In this light, and given the delicate theme of our study, these patients may have experienced a higher threshold to participate.

During the interviews, it was also found that patients often had a difficult time discussing the topics of this study. Many patients seemed to tone down their earlier attribution to jinn during the interviews. More than once they showed to be doubtful about their earlier conviction that jinn had anything to do with their symptoms, or showed to be reluctant to discuss their symptoms and their supposed origins, fearing that the symptoms would thereby increase. We suspect that these reserved reactions were also due to a certain reluctance to share any information on jinn with schooled professionals. In addition, it may have had something to do with a mismatch with the interviewers (all non-Muslim; one Asian, two Caucasian), combined with feelings of fear and shame, which may have contributed to the relatively high number of inconclusive answers (i.e., by 30% of the interviewees). Considering the cautiousness of so many patients, it was in fact surprising that 42% did agree to participate. When asked about their motivation to do so, these patients often expressed the wish to help increase the knowledge of Western mental health professionals about the way they experienced their symptoms, or said that they wanted to do something in return for their therapists. Nonetheless, some of these patients feared stigmatization by third parties to such an extent that they did not dare to speak about their experiences with anyone else.

### Psychopathology

As regards psychopathology, this study demonstrates that patients attributing their mental health problems to jinn are certainly not restricted to those diagnosed (in biomedical terms) with a schizophrenia spectrum disorder. Although the group under study was too small for further statistical analysis, we found that this attribution style cut across many diagnostic categories, varying from anxiety and mood disorders to PTSD and personality disorders. Another remarkable finding was that 87.2% of the interviewees reported having experienced hallucinations at least once in their lives. These hallucinations were mostly auditory in nature, followed by visual and tactile ones, whereas gustatory and olfactory ones were relatively rare. This pattern is in line with the distribution of hallucinations across various sensory modalities as experienced by psychotic patients in Western countries (31, 32), whereas it contradicts the findings from earlier studies on hallucinations in Muslim countries, which found visual hallucinations to be more prevalent (33). An equally remarkable finding was that hallucinations experienced in three or four sensory modalities were far more likely to be attributed to jinn and with a far greater certainty, than those experienced in fewer sensory modalities. An explanation for this might be that multimodal hallucinations tend to have a more life-like character and are, therefore, experienced as more realistic in nature, and/or that these particular hallucinations made a more threatening impression on the patients experiencing them. Whether this explanation is correct should, however, be the subject of further study.

### Idiom of Distress

The present study also confirms the need for adequate knowledge about Muslim explanatory models of disease among biomedically trained health professionals. The explanatory models (and hence the idiom of distress employed) can make differential diagnosis a challenge, especially when health professionals are not used to working with these patients and/or multicultural populations in general. Moreover, additional problems may arise when there is also a language barrier, or when clinical syndromes are not listed in major psychiatric classifications (such as the DSM-5), as is the case with sensed presence and the incubus phenomenon, which may easily be mistaken for symptoms of a schizophrenia spectrum disorder and be treated accordingly (34).

Equally challenging for biomedically trained health professionals is that the Qur’an conceptualizes jinn as living beings, so that patients tend to doubt whether the powers of these beings can be “undone” by medical means. This doubt has substantial consequences for the patient–physician relationship, often leading to ambiguous expectations (at best) regarding what good biomedical treatments can achieve. In the context of the present study, we met a patient who believed that she was haunted by a jinn who wanted to marry her. Feeling thereby deprived of the life she wanted to live, she felt trapped to such an extent that she showed signs of utter helplessness. As a consequence, she dismissed all biomedical interventions indiscriminately, thereby contributing even further to her own feelings of helplessness, as well as of those of all parties involved, including her family. In effect, through her behavior, the jinn was perceived by them as holding her hostage and as playing a role in the family dynamics that was just as real as had a person prevented her from getting married. Similarly, during the course of this study, we found that many patients experienced additional suffering due to the fact that they attributed their symptoms to actual, living beings. They often showed signs of fear and depression while talking about jinn and suffered considerably from the prospect of the consequences that might have.

### Treatment

Many of the patients frequenting our outpatient clinic had previously sought help at general (i.e., non-specialized) mental health services or with faith healers, either in the Netherlands or in their country of origin. Some of them had benefited temporarily from those treatments but, in the end, all of them had felt the need to seek help at our specialized outpatient clinic, often after many years of suffering. But how is one to manage psychiatric symptoms attributed to jinn, the evil eye, or magic in a Western mental-health-care setting, however “specialized” the setting may be? In our experience, sometimes it may be helpful to discuss the existence of various explanatory models of disease, to challenge the notion that the patient’s symptoms are necessarily caused by a jinn and to explain in which ways a biomedical approach might contribute to their well-being. In other cases, it may help to consult an Islamic counselor, preferably in the service of one’s institution, to give due attention to the religious dimension of the patient’s complaints and have explained, if necessary, that the Qur’an does not forbid the undergoing of a biomedical treatment. That said, the question remains whether it suffices to offer medications and psychotherapies “as usual” and relegate religious issues to an Islamic counselor. The principles of cognitive-behavioral therapy (CBT), for example, and those of traditional Islamic healing are divergent (35). Recently, therefore, various initiatives and protocols have been developed in the field of CBT with modifications that are tuned specifically to Islamic values (36, 37). Such modifications are considered important to connect better with patients and thereby promote better treatment compliance, faster recovery, lower rates of relapse, and reduced treatment disparities (37). Like all patient groups, the group of Muslim patients consists of very diverse individuals; however, proper care stands or falls with a broader knowledge of prevailing notions and expectations within the group as a whole and with therapeutic techniques that adhere to the principles of personalized medicine.

### Limitations

A limitation of the present study is the relatively large proportion of eligible patients who declined to participate. Among the various reasons for this, the main ones were the reluctance to discuss the possible role of jinn out of fear for metaphysical repercussions and stigma, and the possible mismatch with the interviewers, none of whom had a Muslim background. While we do advocate the involvement of non-Islamic professionals in studies such as these (if only to create opportunities for a growth of mutual understanding), we recognize that the employment of Muslim researchers might have resulted in a higher number of participants. A second limitation (which follows from the first) is that the numbers were too small for statistical analyses of relations between specific patient characteristics and the propensity to attribute mental health problems to jinn. Therefore, we recommend that future studies be designed in such a way that this power problem can be solved. A third limitation is that this study was carried out in an outpatient population, which makes it difficult to generalize the results to other populations. That said, the present study did succeed in demonstrating that the inclination of Muslim patients to attribute mental health problems to jinn is not confined to psychotic patients alone. A final point that we wish to raise is that the illness attribution of Muslim patients in Western countries is obviously shaped by many more factors, including migration stress, language problems, issues pertaining to the intercultural gap, and cultural conceptions of specific phenomena such as hallucination, dissociation, and derealization, which all deserve to be brought into view if one wishes to obtain a more comprehensive understanding of health issues in this heterogeneous group.

## Conclusion

It is concluded that, in this specialized, psychiatric, transcultural outpatient setting in the Netherlands, 17.7% of Muslim patients attribute their mental health problems to jinn. This underlines the importance of the need for culturally sensitive interviewing techniques for this patient group, including proper attention to attribution styles, religious matters, and system dynamics, as well as the further development of tailor-made therapeutic strategies, preferentially in close collaboration with Islamic counselors.

## Ethics Statement

This study was a pilot study carried out in accordance with the recommendations of the Parnassia Academy, Parnassia Psychiatric Institute, The Hague, with written informed consent from all subjects. All subjects gave written informed consent in accordance with the Declaration of Helsinki. The protocol was approved by the Medical Ethical Committee of University of Leiden, reference number NL44388.058.14.

## Author Contributions

AL contributed to the conception and design of the work, and to the acquisition, analysis, and interpretation of data for the work, drafted and revised the work, gave final approval for the final version to be published, and agreed to be accountable for all aspects of the work in ensuring that questions related to the accuracy or integrity of any part of the work are appropriately investigated and resolved. SG and MD contributed to the analysis and interpretation of data for the work, revised the work, gave final approval for the final version to be published, and agreed to be accountable for all aspects of the work in ensuring that questions related to the accuracy or integrity of any part of the work are appropriately investigated and resolved. HH contributed to the conception and design of the work, and to the analysis and interpretation of data for the work, revised the work, gave final approval for the final version to be published, and agreed to be accountable for all aspects of the work in ensuring that questions related to the accuracy or integrity of any part of the work are appropriately investigated and resolved. JDB contributed to the conception and design of the work, and to the analysis and interpretation of data for the work, drafted and revised the work, gave final approval for the final version to be published, and agreed to be accountable for all aspects of the work in ensuring that questions related to the accuracy or integrity of any part of the work are appropriately investigated and resolved.

## Conflict of Interest Statement

This research was conducted in the absence of any commercial or financial relationships that could be construed as a potential conflict of interest.

## References

[1] Lim A Hoek HW Blom JD. The attribution of psychotic symptoms to jinn in Islamic patients. Transcult Psychiatry (2015) 52:18–32. 10.1177/1363461514543146 25080427

[2] Hassan G Ventevogel P Jefee-Bahloul H Barkli-Oteo A Kirmayer L. Mental health and psychosocial wellbeing of Syrians affected by armed conflict. Epidemiol Psychiatr Sci (2016) 25:129–41. 10.1017/S2045796016000044 26829998 PMC6998596

[3] Khan Qu Sanober A “Jinn possession” and delirious mania in a Pakistani woman. Am J Psychiatry (2016) 173:219–20. 10.1176/appi.ajp.2015.15030281 26926128

[4] Kuittinen S Mölsä M Punamäki RL Tiilikainen M Honkasalo ML. Causal attributions of mental health problems and depressive symptoms among older Somali refugees in Finland. Transcult Psychiatry (2017) 54:211–38. 10.1177/1363461516689003 28398194

[5] Al-Adawi S Dorvlo ASS Al-Ismaily SS Al-Ghafry DA Al-Noobi BZ Al-Salmi A Perception and attitude towards mental illness in Oman. Int J Soc Psychiatry (2002) 48:305–17. 10.1177/002076402128783334 12553410

[6] Al-Habeeb TA. A pilot study of faith healers’ views on evil eye, jinn possession, and magic in the Kingdom of Saudi Arabia. J Family Community Med (2003) 10:31–8. 23012035 PMC3425750

[7] Blom JD Hoffer CBM Djinns. In: Blom JD Sommer IEC, editors. Hallucinations: Research and Practice. New York, NY: Springer (2012). p. 235–47.

[8] Al-Riyami AA Al-Adawi SH Al-Kharusi HA Morsi MM Jaju SS Health services utilization by school going Omani adolescents and youths with DSM-IV mental disorders and barriers to service use. Int J Met Health Syst (2009) 3:22 10.1186/1752-4458-3-22 PMC2761295 19781054

[9] Guthrie EA Abraham S Nawaz S Process of determining the value of belief about jinn possession and whether or not they are a result of mental illness. BMJ Case Rep (2016) 2016 10.1136/bcr-2015-214005 PMC4746541 26838303

[10] Hoffer. [Folk belief and religious healing methods among Muslims in the Netherlands: A historico-sociological analysis of religious-medical thinking and acting]. Thesis in Dutch. Amsterdam: Thela Thesis (2000).

[11] Van Duijl M Nijenhuis E Komproe IH Gernaat HB de Jong IT. Dissociative symptoms and reported trauma among patients with spirit possession and matched healthy controls in Uganda. Cult Med Psychiatry (2010) 34:380–400. 10.1007/s11013-010-9171-1 20401630 PMC2878595

[12] El-Islam M Abu-Dagga SI. Lay explanations of symptoms of mental ill health in Kuwait. Int J Soc Psychiatry (1992) 38:150–6. 10.1177/002076409203800208 1506139

[13] Al-Krenawi A Graham JR Spirit possession and exorcism in the treatment of a Bedouin psychiatric patient. Clin Soc Work J (1997) 25:211–22. 10.1023/A:1025714626136

[14] Bragazzi NL Del Puente G. Panic attacks and possession by djinns: lessons from ethnopsychiatry. Psychol Res Behav Manag (2012) 5:185–90. 10.2147/PRBM.S37714 23293545 PMC3533684

[15] Khalifa N Hardie T Possession and jinn. J R Soc Med (2005) 98:351–3. 10.1177/014107680509800805 16055898 PMC1181833

[16] Finkelstein M. Resource loss, resource gain, PTSD, and dissociation among Ethiopian immigrants in Israel. Scand J Psychol (2016) 57:328–37. 10.1111/sjop.12295 27291081

[17] Selten J-P Veen ND Feller WG Blom JD Schols D Camoenië W Incidence of psychotic disorders in immigrant groups to The Netherlands. Br J Psychiatry (2001) 178:367–72. 10.1192/bjp.178.4.367 11282817

[18] Selten J-P Laan W Veen ND Blom JD Veling W Hoek HW Incidence of schizophrenia among Moroccan immigrants to The Netherlands. Schizophr Res (2010) 124:240–1. 10.1016/j.schres.2010.08.010 20813502

[19] Veling W Susser E van Os J Mackenbach JP Selten J-P Hoek HW. Ethnic density of neighborhoods and incidence of psychotic disorders among immigrants. Am J Psychiatry (2008) 165:66–73. 10.1176/appi.ajp.2007.07030423 18086750

[20] Mullick MSI Khalifa N Nahar JS Alker D-M Beliefs about jinn, black magic and evil eye in Bangladesh: the effects of gender and level of education. Ment Health Relig Cult (2012) 16:719–29. 10.1080/13674676.2012.717918

[21] Shah A Carlsson J [Jinn possession as an explanation of mental illness influences the treatment-seeking behavior]. Article in Danish: Jinn-besaettelse som forklaring på psykisk sygdom har betydning for behandlingssøgende adfaerd. Ugeskrift Laeger (2016) 178:V03160194. 27406054

[22] Aussems C Moslims in Nederland 2014. Utrecht: Kennisplatform Integratie en Samenleving (2016).

[23] Central Bureau of Statistics. (2017). Available from http://statline.cbs.nl/Statweb/publication/?DM=SLNL&PA=82904ned&D1=6&D2=a&D3=a&VW=T

[24] Blom JD Eker H Basalan H Aouaj Y Hoek HW [Hallucinations attributed to jinn]. Article in Dutch: Hallucinaties toegedicht aan djinns. Ned Tijdschr Geneeskd (2010) 154:A973. 20132570

[25] Dein S Alexander M Napier AD. Jinn, psychiatry and contested notions of misfortune among east London Bangladeshis. Transcult Psychiatry (2008) 45:31–55. 10.1177/1363461507087997 18344251

[26] Drieskens B Living with Djinns. Understanding and Dealing with the Invisible in Cairo. Lebanon: Saqi Books (2008).

[27] Hermans P De Wereld van de Djinn. Traditionele Marokkaanse Geneeswijzen. Amsterdam: Bulaaq (2007).

[28] American Psychiatric Association. Diagnostic and Statistical Manual of Mental Disorders, Fourth Edition, Text Revision. Washington, DC: American Psychiatric Association (2000).

[29] Blom JD Alice in wonderland syndrome: a systematic review. Neurol Clin Pract (2016) 6:1–12. 10.1212/CPJ.0000000000000251 27347442 PMC4909520

[30] Brown G Marshall M Bower P Woodham A Waheed W. Barriers to recruiting ethnic minorities to mental health research: a systematic review. Int J Methods Psychiatr Res (2014) 23:36–48. 10.1002/mpr.1434 24474683 PMC6878438

[31] David CN Greenstein D Clasen L Gochman P Miller R Tossell JW Childhood onset schizophrenia: high rate of visual hallucinations. J Am Acad Child Adolesc Psychiatry (2011) 50:681–6. 10.1016/j.jaac.2011.03.020 21703495 PMC3124659

[32] Lim A Hoek HW Deen ML Blom JD GROUP Investigators. Prevalence and classification of hallucinations in multiple sensory modalities in schizophrenia spectrum disorders. Schizophr Res (2016) 176:493–9. 10.1016/j.schres.2016.06.010 27349814

[33] Al-Issa I Social and cultural aspects of hallucinations. Psychol Bull (1977) 3:570–87. 10.1037/0033-2909.84.3.570 859961

[34] Blom JD Psychiatry is warming up to personalized medicine 2.0. Per Med (2017) 14:185–7. 10.2217/pme-2016-0106 29767580

[35] Beshai S Clark CM Dobson KS Conceptual and pragmatic considerations in the use of cognitive-behavioral therapy with Muslim clients. Cognit Ther Res (2013) 37:197–206. 10.1007/s10608-012-9450-y

[36] Van den Berg D Raijmakers B Scholten A [The ghost protocol. Cognitive behavioral therapy in case of ghosts, jinn and magic]. Article in Dutch: The ghost protocol. CGT bij geesten, djinns en magie. Gedragstherapie (2015) 48:222–43.

[37] Husain A Hodge DR Islamically modified cognitive behavioral therapy: Enhancing outcomes by increasing the cultural congruence of cognitive behavioral therapy self-statements. Int Soc Work (2016) 59:393–405. 10.1177/0020872816629193

